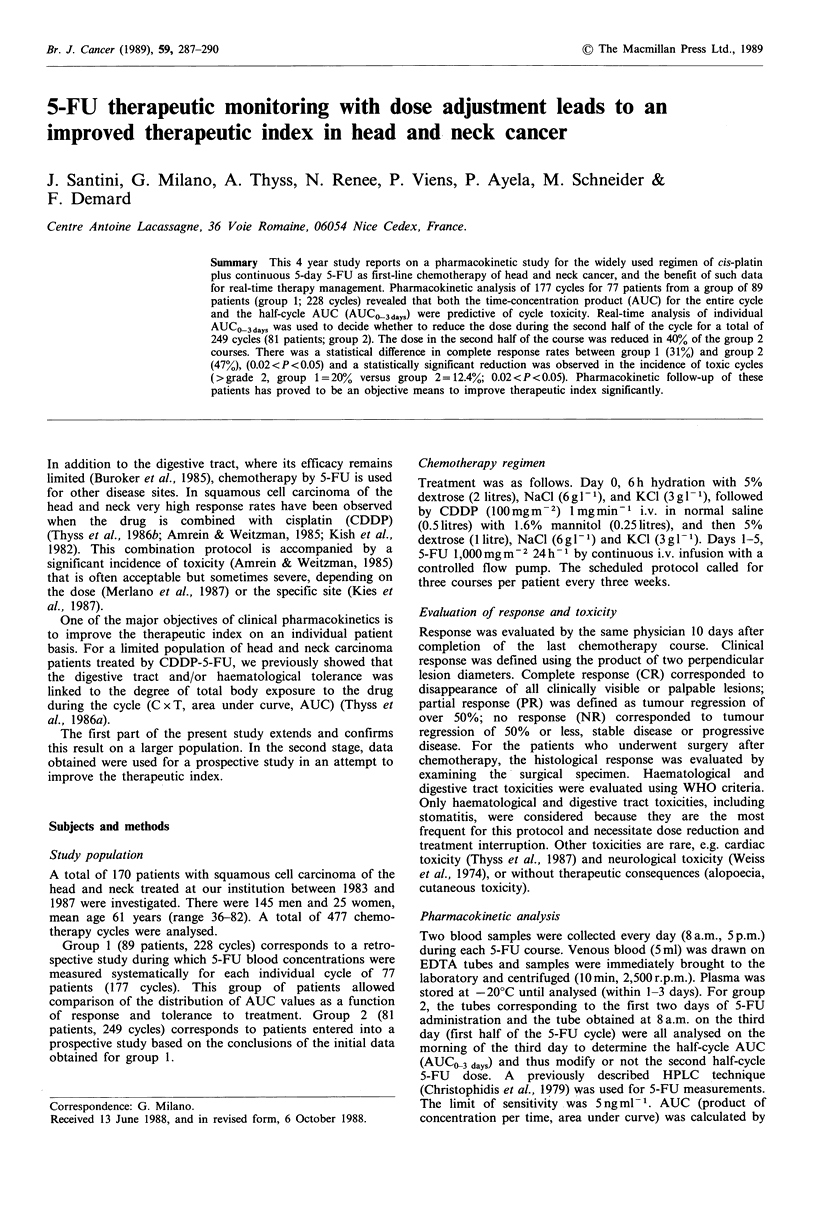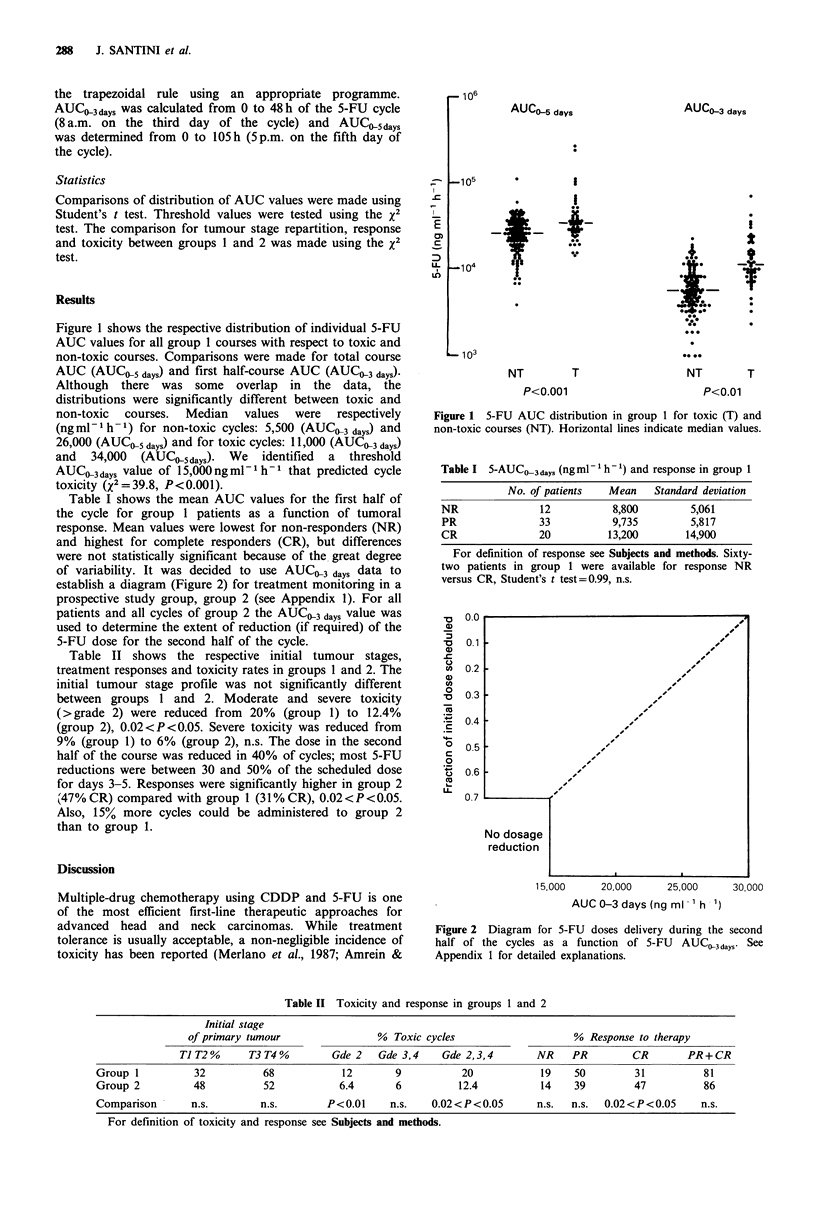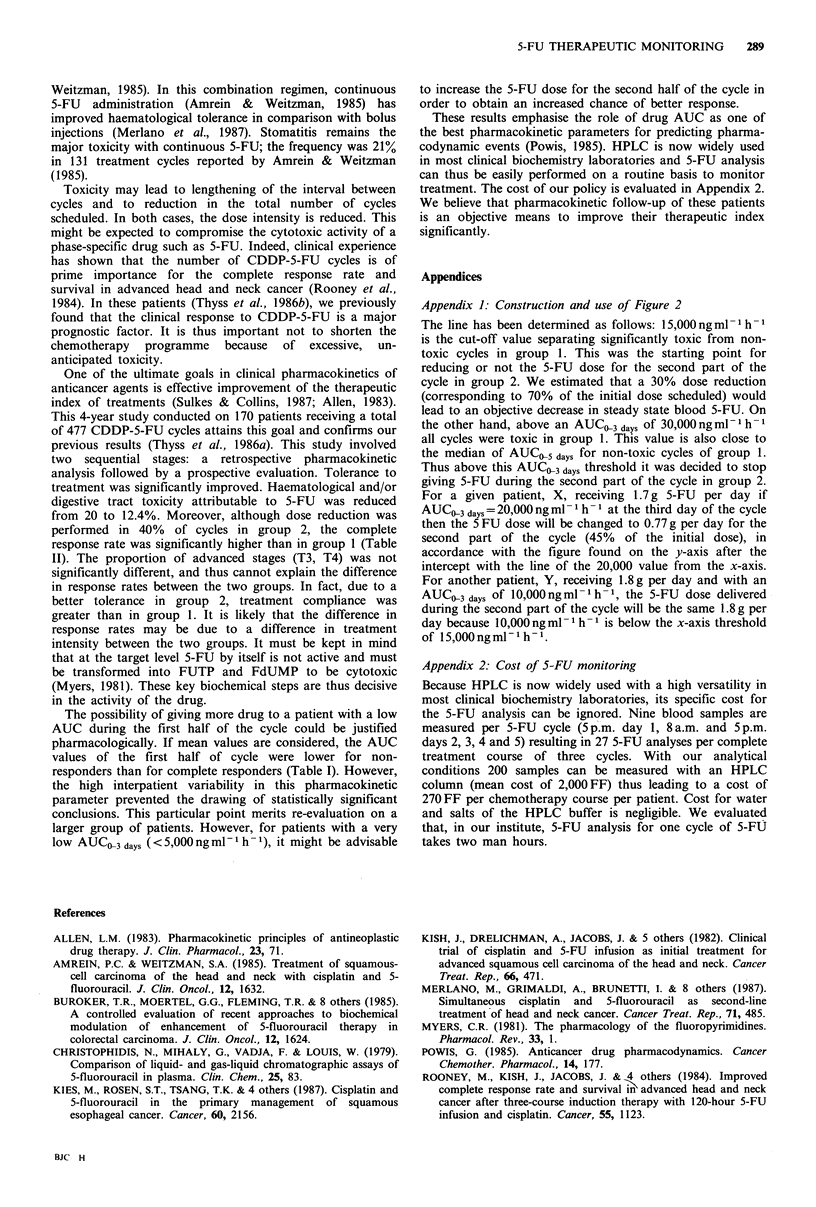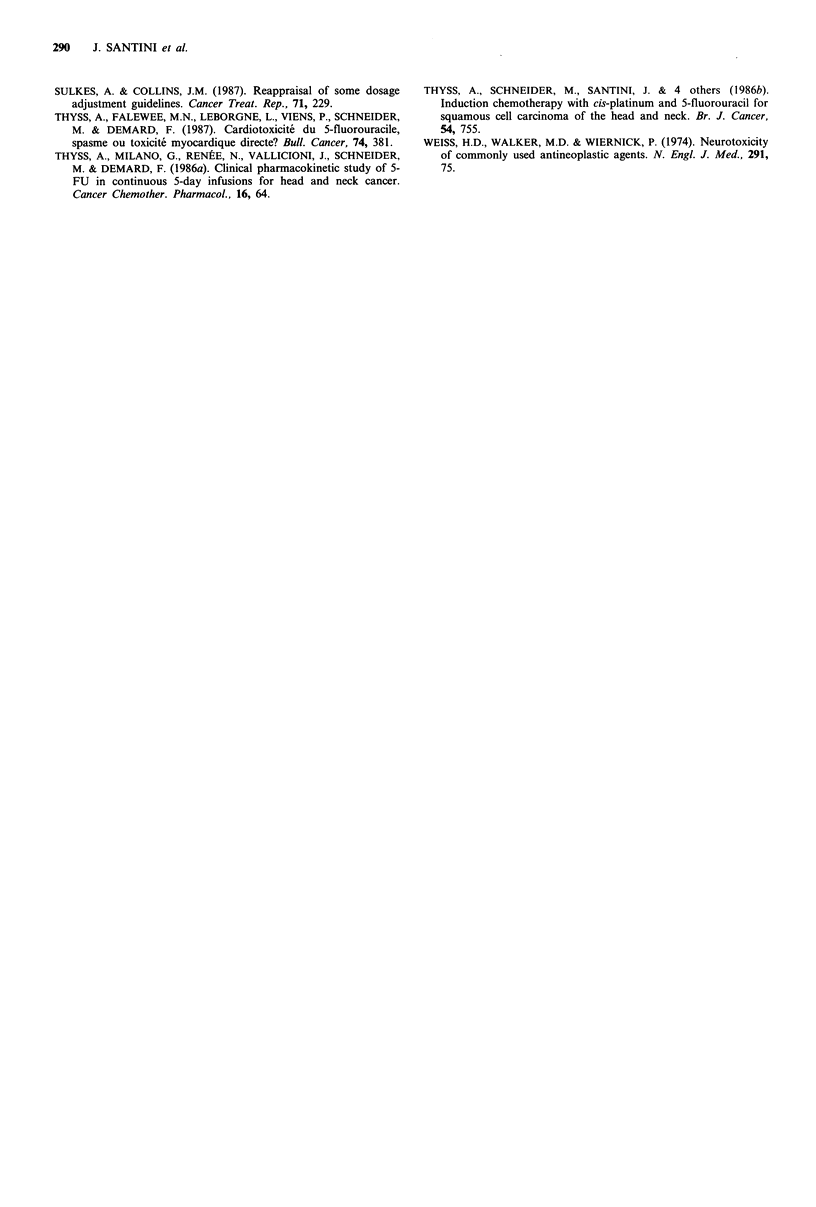# 5-FU therapeutic monitoring with dose adjustment leads to an improved therapeutic index in head and neck cancer.

**DOI:** 10.1038/bjc.1989.59

**Published:** 1989-02

**Authors:** J. Santini, G. Milano, A. Thyss, N. Renee, P. Viens, P. Ayela, M. Schneider, F. Demard

**Affiliations:** Centre Antoine Lacassagne, Nice, France.

## Abstract

This 4 year study reports on a pharmacokinetic study for the widely used regimen of cis-platin plus continuous 5-day 5-FU as first-line chemotherapy of head and neck cancer, and the benefit of such data for real-time therapy management. Pharmacokinetic analysis of 177 cycles for 77 patients from a group of 89 patients (group 1; 228 cycles) revealed that both the time-concentration product (AUC) for the entire cycle and the half-cycle AUC (AUC0-3 days) were predictive of cycle toxicity. Real-time analysis of individual AUC0-3 days was used to decide whether to reduce the dose during the second half of the cycle for a total of 249 cycles (81 patients; group 2). The dose in the second half of the course was reduced in 40% of the group 2 courses. There was a statistical difference in complete response rates between group 1 (31%) and group 2 (47%), (0.02 less than P less than 0.05) and a statistically significant reduction was observed in the incidence of toxic cycles (greater than grade 2, group 1 = 20% versus group 2 = 12.4%; 0.02 less than P less than 0.05). Pharmacokinetic follow-up of these patients has proved to be an objective means to improve therapeutic index significantly.


*Br. J. Cancer* (1989), **59**, 287–290 © The Macmillan Press Ltd., 1989

### 5-FU therapeutic monitoring with dose adjustment leads to an improved therapeutic index in head and neck cancer

J. Santini, G. Milano, A. Thyss, N. Renee, P. Viens, P. Ayela, M. Schneider & F. Demard

*Centre Antoine Lacassagne, 36 Voie Romaine, 06054 Nice Cedex, France.*

**Summary** This 4 year study reports on a pharmacokinetic study for the widely used regimen of *cis*-platin plus continuous 5-day 5-FU as first-line chemotherapy of head and neck cancer, and the benefit of such data for real-time therapy management. Pharmacokinetic analysis of 177 cycles for 77 patients from a group of 89 patients (group 1; 228 cycles) revealed that both the time-concentration product (AUC) for the entire cycle and the half-cycle AUC ($AUC_{0-3\,days}$) were predictive of cycle toxicity. Real-time analysis of individual $AUC_{0-3\,days}$ was used to decide whether to reduce the dose during the second half of the cycle for a total of 249 cycles (81 patients; group 2). The dose in the second half of the course was reduced in 40% of the group 2 courses. There was a statistical difference in complete response rates between group 1 (31%) and group 2 (47%), ($0.02 < P < 0.05$) and a statistically significant reduction was observed in the incidence of toxic cycles (>grade 2, group 1 = 20% versus group 2 = 12.4%; $0.02 < P < 0.05$). Pharmacokinetic follow-up of these patients has proved to be an objective means to improve therapeutic index significantly.

In addition to the digestive tract, where its efficacy remains limited (Buroker *et al.*, 1985), chemotherapy by 5-FU is used for other disease sites. In squamous cell carcinoma of the head and neck very high response rates have been observed when the drug is combined with cisplatin (CDDP) (Thyss *et al.*, 1986*b*; Amrein & Weitzman, 1985; Kish *et al.*, 1982). This combination protocol is accompanied by a significant incidence of toxicity (Amrein & Weitzman, 1985) that is often acceptable but sometimes severe, depending on the dose (Merlano *et al.*, 1987) or the specific site (Kies *et al.*, 1987).

One of the major objectives of clinical pharmacokinetics is to improve the therapeutic index on an individual patient basis. For a limited population of head and neck carcinoma patients treated by CDDP-5-FU, we previously showed that the digestive tract and/or haematological tolerance was linked to the degree of total body exposure to the drug during the cycle (C × T, area under curve, AUC) (Thyss *et al.*, 1986*a*).

The first part of the present study extends and confirms this result on a larger population. In the second stage, data obtained were used for a prospective study in an attempt to improve the therapeutic index.

### Subjects and methods

### *Study population*

A total of 170 patients with squamous cell carcinoma of the head and neck treated at our institution between 1983 and 1987 were investigated. There were 145 men and 25 women, mean age 61 years (range 36–82). A total of 477 chemotherapy cycles were analysed.

Group 1 (89 patients, 228 cycles) corresponds to a retrospective study during which 5-FU blood concentrations were measured systematically for each individual cycle of 77 patients (177 cycles). This group of patients allowed comparison of the distribution of AUC values as a function of response and tolerance to treatment. Group 2 (81 patients, 249 cycles) corresponds to patients entered into a prospective study based on the conclusions of the initial data obtained for group 1.

Correspondence: G. Milano.
Received 13 June 1988, and in revised form, 6 October 1988.

### *Chemotherapy regimen*

Treatment was as follows. Day 0, 6 h hydration with 5% dextrose (2 litres), NaCl ($6\,g\,l^{-1}$), and KCl ($3\,g\,l^{-1}$), followed by CDDP ($100\,mg\,m^{-2}$) $1\,mg\,min^{-1}$ i.v. in normal saline (0.5 litres) with 1.6% mannitol (0.25 litres), and then 5% dextrose (1 litre), NaCl ($6\,g\,l^{-1}$) and KCl ($3\,g\,l^{-1}$). Days 1–5, 5-FU $1{,}000\,mg\,m^{-2}\,24\,h^{-1}$ by continuous i.v. infusion with a controlled flow pump. The scheduled protocol called for three courses per patient every three weeks.

### *Evaluation of response and toxicity*

Response was evaluated by the same physician 10 days after completion of the last chemotherapy course. Clinical response was defined using the product of two perpendicular lesion diameters. Complete response (CR) corresponded to disappearance of all clinically visible or palpable lesions; partial response (PR) was defined as tumour regression of over 50%; no response (NR) corresponded to tumour regression of 50% or less, stable disease or progressive disease. For the patients who underwent surgery after chemotherapy, the histological response was evaluated by examining the surgical specimen. Haematological and digestive tract toxicities were evaluated using WHO criteria. Only haematological and digestive tract toxicities, including stomatitis, were considered because they are the most frequent for this protocol and necessitate dose reduction and treatment interruption. Other toxicities are rare, e.g. cardiac toxicity (Thyss *et al.*, 1987) and neurological toxicity (Weiss *et al.*, 1974), or without therapeutic consequences (alopoecia, cutaneous toxicity).

### *Pharmacokinetic analysis*

Two blood samples were collected every day (8 a.m., 5 p.m.) during each 5-FU course. Venous blood (5 ml) was drawn on EDTA tubes and samples were immediately brought to the laboratory and centrifuged (10 min, 2,500 r.p.m.). Plasma was stored at −20°C until analysed (within 1–3 days). For group 2, the tubes corresponding to the first two days of 5-FU administration and the tube obtained at 8 a.m. on the third day (first half of the 5-FU cycle) were all analysed on the morning of the third day to determine the half-cycle AUC ($AUC_{0-3\,days}$) and thus modify or not the second half-cycle 5-FU dose. A previously described HPLC technique (Christophidis *et al.*, 1979) was used for 5-FU measurements. The limit of sensitivity was $5\,ng\,ml^{-1}$. AUC (product of concentration per time, area under curve) was calculated by the trapezoidal rule using an appropriate programme. $AUC_{0-3\,days}$ was calculated from 0 to 48 h of the 5-FU cycle (8 a.m. on the third day of the cycle) and $AUC_{0-5\,days}$ was determined from 0 to 105 h (5 p.m. on the fifth day of the cycle).

*Statistics*

Comparisons of distribution of AUC values were made using Student's *t* test. Threshold values were tested using the $\chi^2$ test. The comparison for tumour stage repartition, response and toxicity between groups 1 and 2 was made using the $\chi^2$ test.

### Results

Figure 1 shows the respective distribution of individual 5-FU AUC values for all group 1 courses with respect to toxic and non-toxic courses. Comparisons were made for total course AUC ($AUC_{0-5\,days}$) and first half-course AUC ($AUC_{0-3\,days}$). Although there was some overlap in the data, the distributions were significantly different between toxic and non-toxic courses. Median values were respectively ($ng\,ml^{-1}\,h^{-1}$) for non-toxic cycles: 5,500 ($AUC_{0-3\,days}$) and 26,000 ($AUC_{0-5\,days}$) and for toxic cycles: 11,000 ($AUC_{0-3\,days}$) and 34,000 ($AUC_{0-5\,days}$). We identified a threshold $AUC_{0-3\,days}$ value of 15,000 $ng\,ml^{-1}\,h^{-1}$ that predicted cycle toxicity ($\chi^2 = 39.8$, $P < 0.001$).

Table I shows the mean AUC values for the first half of the cycle for group 1 patients as a function of tumoral response. Mean values were lowest for non-responders (NR) and highest for complete responders (CR), but differences were not statistically significant because of the great degree of variability. It was decided to use $AUC_{0-3\,days}$ data to establish a diagram (Figure 2) for treatment monitoring in a prospective study group, group 2 (see Appendix 1). For all patients and all cycles of group 2 the $AUC_{0-3\,days}$ value was used to determine the extent of reduction (if required) of the 5-FU dose for the second half of the cycle.

Table II shows the respective initial tumour stages, treatment responses and toxicity rates in groups 1 and 2. The initial tumour stage profile was not significantly different between groups 1 and 2. Moderate and severe toxicity (>grade 2) were reduced from 20% (group 1) to 12.4% (group 2), $0.02 < P < 0.05$. Severe toxicity was reduced from 9% (group 1) to 6% (group 2), n.s. The dose in the second half of the course was reduced in 40% of cycles; most 5-FU reductions were between 30 and 50% of the scheduled dose for days 3–5. Responses were significantly higher in group 2 (47% CR) compared with group 1 (31% CR), $0.02 < P < 0.05$. Also, 15% more cycles could be administered to group 2 than to group 1.

### Discussion

Multiple-drug chemotherapy using CDDP and 5-FU is one of the most efficient first-line therapeutic approaches for advanced head and neck carcinomas. While treatment tolerance is usually acceptable, a non-negligible incidence of toxicity has been reported (Merlano *et al.*, 1987; Amrein &

**Figure 1** 5-FU AUC distribution in group 1 for toxic (T) and non-toxic courses (NT). Horizontal lines indicate median values.

**Table I** 5-$AUC_{0-3\,days}$ ($ng\,ml^{-1}\,h^{-1}$) and response in group 1

| | *No. of patients* | *Mean* | *Standard deviation* |
|---|---|---|---|
| NR | 12 | 8,800 | 5,061 |
| PR | 33 | 9,735 | 5,817 |
| CR | 20 | 13,200 | 14,900 |

For definition of response see **Subjects and methods**. Sixty-two patients in group 1 were available for response NR versus CR, Student's *t* test = 0.99, n.s.

**Figure 2** Diagram for 5-FU doses delivery during the second half of the cycles as a function of 5-FU $AUC_{0-3\,days}$. See Appendix 1 for detailed explanations.

**Table II** Toxicity and response in groups 1 and 2

| | *Initial stage of primary tumour* | | *% Toxic cycles* | | | *% Response to therapy* | | | |
|---|---|---|---|---|---|---|---|---|---|
| | *T1 T2 %* | *T3 T4 %* | *Gde 2* | *Gde 3,4* | *Gde 2,3,4* | *NR* | *PR* | *CR* | *PR+CR* |
| Group 1 | 32 | 68 | 12 | 9 | 20 | 19 | 50 | 31 | 81 |
| Group 2 | 48 | 52 | 6.4 | 6 | 12.4 | 14 | 39 | 47 | 86 |
| Comparison | n.s. | n.s. | $P<0.01$ | n.s. | $0.02<P<0.05$ | n.s. | n.s. | $0.02<P<0.05$ | n.s. |

For definition of toxicity and response see **Subjects and methods**.

Weitzman, 1985). In this combination regimen, continuous 5-FU administration (Amrein & Weitzman, 1985) has improved haematological tolerance in comparison with bolus injections (Merlano *et al.*, 1987). Stomatitis remains the major toxicity with continuous 5-FU; the frequency was 21% in 131 treatment cycles reported by Amrein & Weitzman (1985).

Toxicity may lead to lengthening of the interval between cycles and to reduction in the total number of cycles scheduled. In both cases, the dose intensity is reduced. This might be expected to compromise the cytotoxic activity of a phase-specific drug such as 5-FU. Indeed, clinical experience has shown that the number of CDDP-5-FU cycles is of prime importance for the complete response rate and survival in advanced head and neck cancer (Rooney *et al.*, 1984). In these patients (Thyss *et al.*, 1986*b*), we previously found that the clinical response to CDDP-5-FU is a major prognostic factor. It is thus important not to shorten the chemotherapy programme because of excessive, unanticipated toxicity.

One of the ultimate goals in clinical pharmacokinetics of anticancer agents is effective improvement of the therapeutic index of treatments (Sulkes & Collins, 1987; Allen, 1983). This 4-year study conducted on 170 patients receiving a total of 477 CDDP-5-FU cycles attains this goal and confirms our previous results (Thyss *et al.*, 1986*a*). This study involved two sequential stages: a retrospective pharmacokinetic analysis followed by a prospective evaluation. Tolerance to treatment was significantly improved. Haematological and/or digestive tract toxicity attributable to 5-FU was reduced from 20 to 12.4%. Moreover, although dose reduction was performed in 40% of cycles in group 2, the complete response rate was significantly higher than in group 1 (Table II). The proportion of advanced stages (T3, T4) was not significantly different, and thus cannot explain the difference in response rates between the two groups. In fact, due to a better tolerance in group 2, treatment compliance was greater than in group 1. It is likely that the difference in response rates may be due to a difference in treatment intensity between the two groups. It must be kept in mind that at the target level 5-FU by itself is not active and must be transformed into FUTP and FdUMP to be cytotoxic (Myers, 1981). These key biochemical steps are thus decisive in the activity of the drug.

The possibility of giving more drug to a patient with a low AUC during the first half of the cycle could be justified pharmacologically. If mean values are considered, the AUC values of the first half of cycle were lower for non-responders than for complete responders (Table I). However, the high interpatient variability in this pharmacokinetic parameter prevented the drawing of statistically significant conclusions. This particular point merits re-evaluation on a larger group of patients. However, for patients with a very low $AUC_{0-3\ days}$ ($<5{,}000\ ng\ ml^{-1}\ h^{-1}$), it might be advisable to increase the 5-FU dose for the second half of the cycle in order to obtain an increased chance of better response.

These results emphasise the role of drug AUC as one of the best pharmacokinetic parameters for predicting pharmacodynamic events (Powis, 1985). HPLC is now widely used in most clinical biochemistry laboratories and 5-FU analysis can thus be easily performed on a routine basis to monitor treatment. The cost of our policy is evaluated in Appendix 2. We believe that pharmacokinetic follow-up of these patients is an objective means to improve their therapeutic index significantly.

### Appendices

### *Appendix 1: Construction and use of Figure 2*

The line has been determined as follows: $15{,}000\ ng\ ml^{-1}\ h^{-1}$ is the cut-off value separating significantly toxic from non-toxic cycles in group 1. This was the starting point for reducing or not the 5-FU dose for the second part of the cycle in group 2. We estimated that a 30% dose reduction (corresponding to 70% of the initial dose scheduled) would lead to an objective decrease in steady state blood 5-FU. On the other hand, above an $AUC_{0-3\ days}$ of $30{,}000\ ng\ ml^{-1}\ h^{-1}$ all cycles were toxic in group 1. This value is also close to the median of $AUC_{0-5\ days}$ for non-toxic cycles of group 1. Thus above this $AUC_{0-3\ days}$ threshold it was decided to stop giving 5-FU during the second part of the cycle in group 2. For a given patient, X, receiving 1.7 g 5-FU per day if $AUC_{0-3\ days} = 20{,}000\ ng\ ml^{-1}\ h^{-1}$ at the third day of the cycle then the 5FU dose will be changed to 0.77 g per day for the second part of the cycle (45% of the initial dose), in accordance with the figure found on the *y*-axis after the intercept with the line of the 20,000 value from the *x*-axis. For another patient, Y, receiving 1.8 g per day and with an $AUC_{0-3\ days}$ of $10{,}000\ ng\ ml^{-1}\ h^{-1}$, the 5-FU dose delivered during the second part of the cycle will be the same 1.8 g per day because $10{,}000\ ng\ ml^{-1}\ h^{-1}$ is below the *x*-axis threshold of $15{,}000\ ng\ ml^{-1}\ h^{-1}$.

### *Appendix 2: Cost of 5-FU monitoring*

Because HPLC is now widely used with a high versatility in most clinical biochemistry laboratories, its specific cost for the 5-FU analysis can be ignored. Nine blood samples are measured per 5-FU cycle (5 p.m. day 1, 8 a.m. and 5 p.m. days 2, 3, 4 and 5) resulting in 27 5-FU analyses per complete treatment course of three cycles. With our analytical conditions 200 samples can be measured with an HPLC column (mean cost of 2,000 FF) thus leading to a cost of 270 FF per chemotherapy course per patient. Cost for water and salts of the HPLC buffer is negligible. We evaluated that, in our institute, 5-FU analysis for one cycle of 5-FU takes two man hours.

### References

ALLEN, L.M. (1983). Pharmacokinetic principles of antineoplastic drug therapy. *J. Clin. Pharmacol.*, **23**, 71.

AMREIN, P.C. & WEITZMAN, S.A. (1985). Treatment of squamous-cell carcinoma of the head and neck with cisplatin and 5-fluorouracil. *J. Clin. Oncol.*, **12**, 1632.

BUROKER, T.R., MOERTEL, G.G., FLEMING, T.R. & 8 others (1985). A controlled evaluation of recent approaches to biochemical modulation of enhancement of 5-fluorouracil therapy in colorectal carcinoma. *J. Clin. Oncol.*, **12**, 1624.

CHRISTOPHIDIS, N., MIHALY, G., VADJA, F. & LOUIS, W. (1979). Comparison of liquid- and gas-liquid chromatographic assays of 5-fluorouracil in plasma. *Clin. Chem.*, **25**, 83.

KIES, M., ROSEN, S.T., TSANG, T.K. & 4 others (1987). Cisplatin and 5-fluorouracil in the primary management of squamous esophageal cancer. *Cancer*, **60**, 2156.

KISH, J., DRELICHMAN, A., JACOBS, J. & 5 others (1982). Clinical trial of cisplatin and 5-FU infusion as initial treatment for advanced squamous cell carcinoma of the head and neck. *Cancer Treat. Rep.*, **66**, 471.

MERLANO, M., GRIMALDI, A., BRUNETTI, I. & 8 others (1987). Simultaneous cisplatin and 5-fluorouracil as second-line treatment of head and neck cancer. *Cancer Treat. Rep.*, **71**, 485.

MYERS, C.R. (1981). The pharmacology of the fluoropyrimidines. *Pharmacol. Rev.*, **33**, 1.

POWIS, G. (1985). Anticancer drug pharmacodynamics. *Cancer Chemother. Pharmacol.*, **14**, 177.

ROONEY, M., KISH, J., JACOBS, J. & 4 others (1984). Improved complete response rate and survival in advanced head and neck cancer after three-course induction therapy with 120-hour 5-FU infusion and cisplatin. *Cancer*, **55**, 1123.

SULKES, A. & COLLINS, J.M. (1987). Reappraisal of some dosage adjustment guidelines. *Cancer Treat. Rep.*, **71**, 229.

THYSS, A., FALEWEE, M.N., LEBORGNE, L., VIENS, P., SCHNEIDER, M. & DEMARD, F. (1987). Cardiotoxicité du 5-fluorouracile, spasme ou toxicité myocardique directe? *Bull. Cancer*, **74**, 381.

THYSS, A., MILANO, G., RENÉE, N., VALLICIONI, J., SCHNEIDER, M. & DEMARD, F. (1986*a*). Clinical pharmacokinetic study of 5-FU in continuous 5-day infusions for head and neck cancer. *Cancer Chemother. Pharmacol.*, **16**, 64.

THYSS, A., SCHNEIDER, M., SANTINI, J. & 4 others (1986*b*). Induction chemotherapy with *cis*-platinum and 5-fluorouracil for squamous cell carcinoma of the head and neck. *Br. J. Cancer*, **54**, 755.

WEISS, H.D., WALKER, M.D. & WIERNICK, P. (1974). Neurotoxicity of commonly used antineoplastic agents. *N. Engl. J. Med.*, **291**, 75.